# Seasonal Changes in the Metabolic Profiles and Biological Activity in Leaves of *Diospyros digyna* and *D. rekoi* “Zapote” Trees

**DOI:** 10.3390/plants8110449

**Published:** 2019-10-25

**Authors:** Ernesto Ramírez-Briones, Ramón Rodríguez-Macías, Eduardo Salcedo-Pérez, Enrique Ramírez-Chávez, Jorge Molina-Torres, Axel Tiessen, José Ordaz-Ortiz, Norma Martínez-Gallardo, John P. Délano-Frier, Julia Zañudo-Hernández

**Affiliations:** 1 Department of Ecology, Centro Universitario de Ciencias Biológicas y Agropecuarias, Camino Ing. Ramón Padilla Sánchez No. 2100, La Venta del Astillero, Zapopan 45110, Jalisco, Mexico; ernestor.briones@gmail.com; 2 Department of Botany and Zoology, Centro Universitario de Ciencias Exactas e Ingeniería, Camino Ing. Ramón Padilla Sánchez No. 2100, La Venta del Astillero, Zapopan 45110, Jalisco, Mexico; ramon.rmacias@academicos.udg.mx (R.R.-M.); esalcedoperez@yahoo.com (E.S.-P.); 3 Department of Biotechnology and Biochemistry, Centro de Investigación y de Estudios Avanzados del Instituto Politécnico Nacional, Unidad Irapuato, Libramiento Norte Carretera Irapuato León Kilómetro 9.6, Carretera Irapuato León, Irapuato 36824, Guanajuato, Mexico; enrique.ramirez@cinvestav.mx (E.R.-C.); jorge.molina@cinvestav.mx (J.M.-T.); axel.tiessen@cinvestav.mx (A.T.); norma.martinez@cinvestav.mx (N.M.-G.); 4 Metabolomics Laboratory, Centro de Investigación y de Estudios Avanzados del Instituto Politécnico Nacional, Unidad de Genómica Avanzada, Libramiento Norte Carretera Irapuato León Kilómetro 9.6, Carretera Irapuato León, Irapuato 36824, Guanajuato, Mexico; jose.ordaz.ortiz@cinvestav.mx

**Keywords:** Antioxidant capacity, *Diospyros*, flavonoid glycosides, metabolic fingerprints, phenolics, secondary metabolites

## Abstract

Leaves of semi-domesticated *Diospyros digyna* and wild *D. rekoi* trees, sampled seasonally in Mexico in 2014, were analyzed. Metabolic fingerprints revealed higher metabolite diversity in *D. rekoi* leaves. The TLC bands characteristic of glycosylated flavonoids, predominant in this species, matched the detection of quercetin and quercetin 3-O-glucuronides by liquid chromatography (UPLC-MS) of spring leaf extracts (LEs). Further gas chromatography (GC-MS) analysis revealed abundant fatty acids, organic acids, and secondary metabolites including trigonelline, p-coumaric, and ferulic and nicotinic acids. Phenolic-like compounds prevailed in *D. digyna* LEs, while unidentified triterpenoids and dihydroxylated coumarins were detected by UPLC-MS and GC-MS. A paucity of leaf metabolites in leaves of this species, compared to *D. rekoi*, was evident. Higher antioxidant capacity (AOC) was detected in *D. digyna* LEs. The AOC was season-independent in *D. digyna* but not in *D. rekoi*. The AOC in both species was concentrated in distinct TLC single bands, although seasonal variation in band intensity was observed among trees sampled. The AOC in *D. digyna* LEs could be ascribed to the coumarin esculetin. The LEs moderately inhibited phytopathogenic bacteria but not fungi. Leaf chemistry differences in these Mesoamerican *Diospyros* species substantiated previous variability reported in tree physiology and fruit physical chemistry, postulated to result from domestication and seasonality.

## 1. Introduction

The abundant plant diversity characteristic of Mexico represents a potentially rich source of secondary metabolites having bioactive properties. Unfortunately, a significant part of indigenous knowledge about their various beneficial effects is believed to have been lost as the Spanish domination gradually consolidated [1,2]. The importance of such plants for many of these cultures, based on their quotidian use, either as a source of food, a curative agent, and/or a communication channel with the divine, was, in many cases, profound. This was manifested, for example, in the assignment of town names, many of which reflect the value that certain plants represented for these people. A relevant example is the frequency with which towns called “Zapotlán” occur in certain regions of western Mexico. This refers to the abundance of the characteristic “Zapote” (*Diospyros* spp.) trees, known as “Tlilzapotl” in the Nahuatl language, which is still relevant in certain regional habitats where they still flourish [3]. 

Mexico represents an important region of diversity for the *Diospyros* genus, where more than 20 different species are found, mostly in tropical and subtropical climes, but also sporadically in temperate, mountainous regions [4]. Trees of these species do not grow profusely, being usually found in low population densities. They are appreciated, however, for the quality of their fruits, which are employed as a food source by the indigenous population [5]. In western Mexico, in which at least six *Diopyros* species are endemic to it, *D. digyna* Jacq. excels for its fruit production, due partly to a domestication process and more organized cultural practices. Fruits are not only consumed fresh, but are used in the elaboration of juice, ice creams, jellies, alcoholic beverages, and certain traditional foods, such as “moles”. The latter has contributed to their worldwide commercialization as an “exotic” fruit [6], sought not only as a food source, but as a remedy for several ailments, a property shared with several other *Diospyros* species and which has been validated by a growing body of experimental evidence [6,7,8,9,10,11]. Contrarily, the fruits of the poorly known *D. rekoi* Standl., although considered to be equally or more savory than *D. digyna* fruits, are collected from trees growing in the wild, usually by shepherds and other itinerant people (E. Ramírez-Briones, personal communication).

Multiple studies have concentrated on the study of *Diospyros* plant secondary metabolites for a diversity of purposes. Flavonoids, polyphenols, phenolic acids coumarins, anthocyanins, terpenes, tannins, and naphthoquinones, isolated from practically all its organs, have been shown to have possible antitumor, analgesic, anti-inflammatory, antiviral and, antioxidant properties, among others [6,7,8,9,11,12,13]. However, it is important to note that, apart from the pioneer study by Yahia et al. [6], followed shortly after by a report by Moo-Huchin et al. [14], the physiology and metabolic composition of Mesoamerican *Diospyros* species has been scarcely studied. Nevertheless, similar to other better-studied species, such as *D. kaki* [13], they have also been found to be a rich source of antioxidants, due to the fact of their high content of polyphenols, flavonoids, and anthocyanins, in addition to carotenoids and tocopherols. 

This study represents part of an ongoing investigation to determine the influence that domestication, seasonality, growing habitat, and cultural practices, or the lack thereof, have on the physiology and metabolism of two Mesoamerican *Diospyros* species: *D. digyna* and *D. rekoi*. Similar to previous reports describing significant differences in tree physiology [15] and fruit physicochemical properties [16], the present investigation revealed that metabolic abundance and diversity was greater in leaves of wild *D. rekoi* trees compared to those of domesticated *D. digyna*. This finding suggests that the domestication process in *D. digyna* reduced the metabolic richness of their leaves without affecting important nutraceutical traits, such as antioxidant capacity. In addition, the leaf chemistry was affected by seasonality in a species-specific way. The above results also imply that wild Mesoamerican species, including not only *D. rekoi* but possibly members of the *D. salicifolia* complex [17] represent a potential source of novel bioactive compounds that could be used for breeding purposes. This information could also encourage the cultivation of *D. digyna* not only as a source of fruits but of medicinal leaves, as well, and the in situ conservation and commercial exploitation of *D. rekoi* and other undomesticated Mesoamerican species. 

## 2. Results

**Figure 1:**
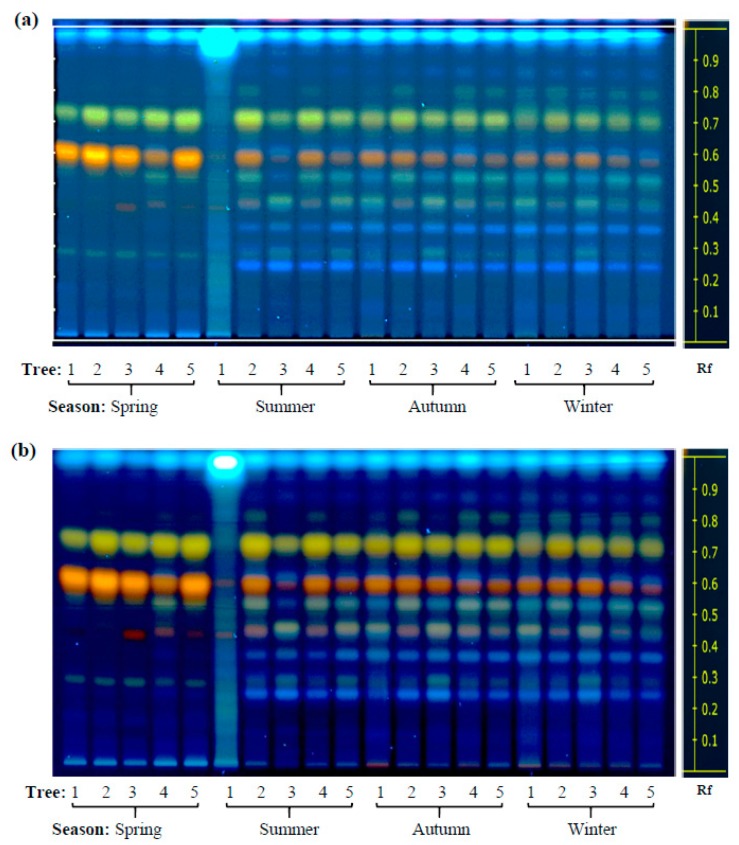
Seasonal variation in the content of phenolic secondary metabolites in *Diospyros rekoi*. High performance-thin layer chromatography (HP-TLC) traces of *D. rekoi* leaf extracts visualized under (**a**) UV light (254 nm) and (**b**) UV light (366 nm) after derivatization with the NP/PEG reagent. Lanes: 1–5 represent leaf extracts from five trees (1–5) that were systematically sampled in the spring, summer, autumn, and winter of 2014. Band Rf values are represented on the right-side end of the figures.

**Figure 2:**
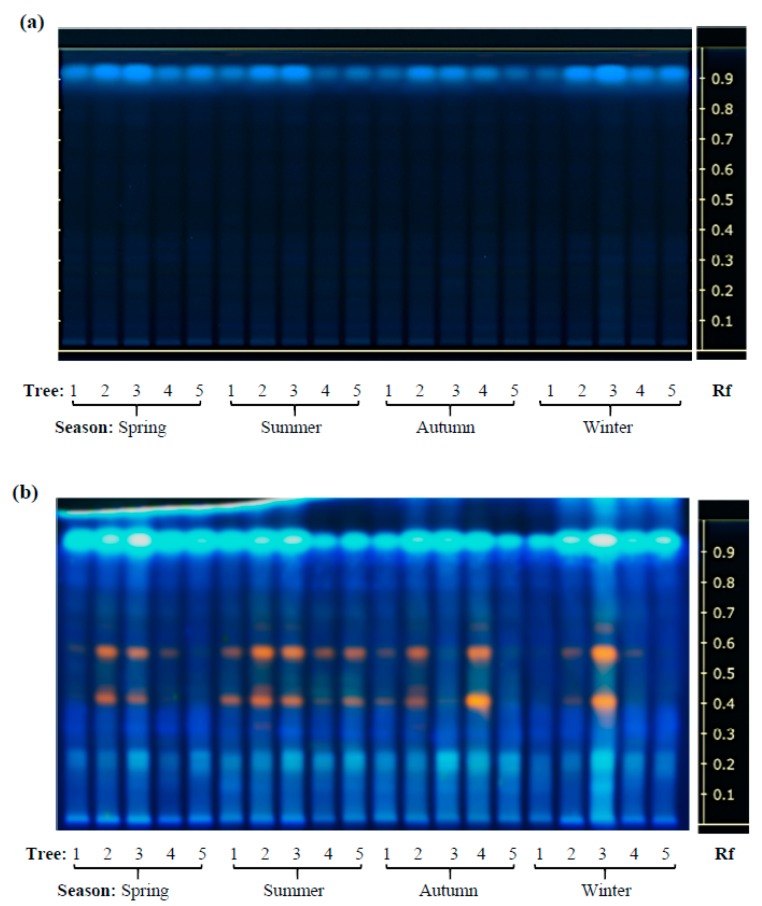
Seasonal variation in the content of phenolic secondary metabolites in *Diospyros digyna*. The HP-TLC traces of *D. digyna* leaf extracts visualized under (**a**) UV light (254 nm) and (**b**) UV light (366 nm) after derivatization with the NP/PEG reagent. Lanes: 1–5 represent leaf extracts from five trees (1–5) that were systematically sampled in the spring, summer, autumn, and winter of 2014. Band Rf values are represented on the right-side end of the figure.

High performance thin layer chromatography (HP-TLC) was initially used in this study to define the phytochemical profile and antioxidant activity of leaves of two contrasting *Diospyros* tree species sampled in different locations of western Mexico during a 16-month interval that included the duration of 2014 [15]. Based on previously published findings [9,12,13], the HP-TLC runs focused on the analysis of phenol and flavonoid compounds which were found to accumulate in the leaf extracts (LEs) of these trees in both a species- and season-dependent manner, as shown in Figure 1 and Figure 2. The accumulation of certain secondary metabolites was more intense in LEs of *D. rekoi* trees sampled in the spring of 2014, although their gradual decrease in intensity observed later in the year was accompanied by a greater metabolite diversity (Figure 1; Table S1). The metabolic profile shown by *D. digyna* LEs was less diversified and did not show evident season-related variations in abundance or complexity (Figure 2). The use of the NP reagent (Figure 1b and Figure 2b) permitted a better visualization of the compounds. Thus, the orange–yellow bands were more prevalent in the HP-TLC traces produced by *D. rekoi* LEs (Figure 1), mostly in those obtained from trees sampled in the spring. Contrariwise, bands having the characteristic blue coloration produced by polyphenols or coumarins, which migrated within a 0.2 ≥ Rf ≤ 0.6 range, were weak to undetectable in *D. rekoi* spring LEs but accumulated from the summer onwards (Figure 1). A blue band migrating very close to the solvent front (Rf ≥ 0.9) was dominant in *D. digyna* LEs (Figure 2) which produced less complex but more consistent band patterns throughout the year. 

**Figure 3:**
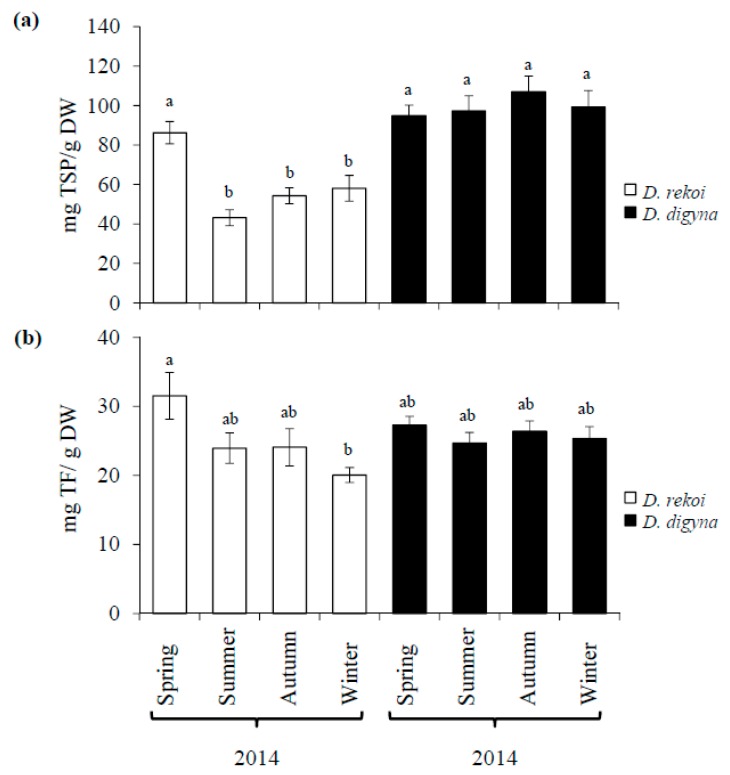
Seasonal variation of total soluble phenols (TSPs) and flavonoids (TFs) in leaves of *Diospyros digyna and D. rekoi*. Average seasonal variation in (**a**) TSPs and (**b**) TFs, expressed as caffeic acid and catechin equivalents, respectively, was determined in vitro in 60% aqueous methanolic leaf extracts of *D. digyna* and *D. rekoi* trees. The bars represent the mean values obtained from leaf extracts produced from the pooled leaves of five trees sampled in the spring, summer, autumn, and winter of 2014, respectively. Intervals over the bars represent the standard error of the means, whereas different letters over the bars represent statistically different values at *p* ≤ 0.05 (Tukey–Kramer test). DW = dry weight.

Total soluble phenol (TSP) content was significantly higher in *D. digyna* LEs (Figure 3a) and remained relatively constant irrespective of the sampling season. Conversely, TSP in *D. rekoi* LEs, in accordance with the HP-TLC data in Figure 1, was significantly higher in spring-sampled leaves. Apart from the significant difference in total flavonoids (TFs) detected between spring (highest) and winter (lowest) *D. rekoi* LEs, the TF content was similar in LEs of both species (Figure 3b). A complementary UPLC/MS analysis of *D. digyna* and *D. rekoi* LEs sampled in this season partly coincided with the differences described above, as shown by the PCA (Figure S1) and S-Plot data (Figure S2). Highly discriminant metabolites detected among species are listed in Table 1. Among the latter, 4, 7-dihydroxycoumarin and two unidentified triterpenes were significantly more abundant in spring *D. digyna* leaves, whereas flavonols, such as quercetin, quercetin-3′-glucuronides, and 3, 4, 5, 7, 3′, 4′, 5′-heptahydroxyflavan (or leucodelphinidin), significantly accumulated to higher levels in spring LEs of *D. rekoi*. In addition, GC-MS analysis of LEs from each season of 2014 supported the above findings by showing a higher abundance and diversity of metabolites in *D. rekoi* LEs compared to *D. digyna* (Table 2; Table S1). Seasonal effects were apparent in the former species, since lower metabolite abundance and diversity was detected in spring and, predominantly, summer LEs (Table S1). Species-specific accumulation of certain metabolites was also detected: trigonelline, p-coumaric, and ferulic acids were only detected in *D. rekoi* LEs, whereas 6, 7 dihydroxycoumarin was exclusively found in *D. digyna* LEs. Although nicotinic and p-hydroxybenzoic acids were found in both species; they were more abundant in LEs of *D. rekoi*. Organic acids and free fatty acids (FAs) were also more abundant in *D. rekoi* LEs which also accumulated distinct short chain FAs that were absent in *D. digyna*.

**Figure 4:**
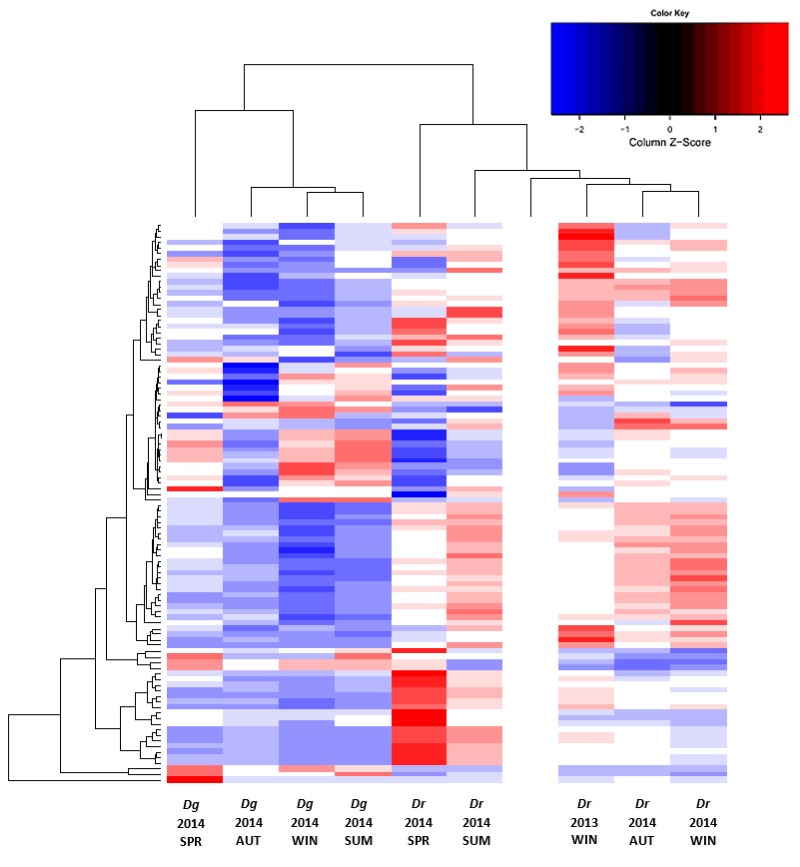
Seasonal metabolic diversity in leaves of *D. digyna* (*Dg*) and *D. rekoi* (*Dr*). The metabolic heat map was obtained from acidified methanol extracts obtained from leaves collected from *Dg* and *Dr* trees at different seasons: SPR, spring 2014; SUM, summer 2014; AUT, autumn 2014, and WIN, winter, 2013 and 2014 (for (*Dr*) and 2014 (*Dg*)). The 100 most abundant ionizable metabolites were selected to obtain the metabolic heat map within an 80–1300 m/z range. All measurements were performed in triplicate. The color-coded matrix elements and adjacent dendrograms indicate the functional relationships among the following variables: 100 ionizable metabolites’ abundance, detected by untargeted direct-injection electrospray mass spectrometry (DIESI-MS) analysis; two *Diospyros* tree species; and five seasons. The blank column represents a solvent control used to calibrate the system.

The untargeted metabolic analysis included more than 900 ions detected in positive ionization mode. A heat map constructed with the 100 most abundant ions concurred with the metabolomic variability and abundance observed between LEs of *D. digyna* and *D. rekoi* described above (Figure 4). It also indicated a clear separation between species and seasons. Thus, *D. rekoi* leaf extracts sampled in the spring and summer of 2014, clustered in the same dendrogram of the heat map, apart from those obtained in the autumn and winter. This orderly warm-to-cold seasonal transition was not apparent in the metabolic ion patterns of *D. digyna* LEs. This was consistent with their lack of seasonal variation, as observed in the HP-TLC traces and in the TSP and TF analyses, respectively.

Slight differences in leaf secondary metabolite accumulation patterns were also observed among individual trees. For instance, the band pattern shown in the LEs of *D. rekoi* tree 1 (T1) in the summer was more akin to those characteristic of *D. dygina* LEs, with a strong signal corresponding to a blue-band phenolic compound migrating very near the solvent front (Rf ≥ 0.9) and a weakened intensity of the prominent orange–yellow bands characteristic of *D. rekoi* LEs (Figure 1b). A similar Rf ≥ 0.9 band, together with orange-colored bands migrating between 0.4 ≥ Rf ≤ 0.6, also had a tendency to be consistently weaker in LEs of *D. digyna* T1 tree, irrespective of the season examined (Figure 2).

**Figure 5:**
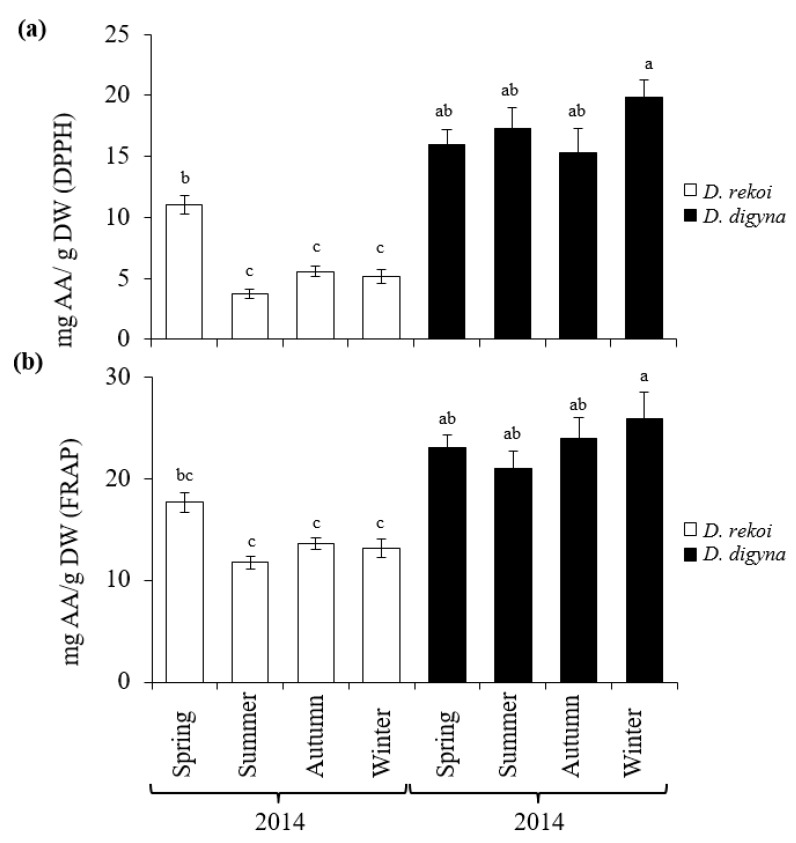
Average seasonal variation in antioxidant activities in aqueous methanol leaf extracts sampled from *Diospyros digyna and D. rekoi* trees. Antioxidant activity was measured using the (**a**) DPPH (2, 2′-diphenyl-1-picrylhydrazyl) and (**b**) FRAP (ferric ion-reducing antioxidant power) assays. Both were expressed as ascorbic acid (AA) equivalents. The bars represent the mean values obtained from leaf extracts produced from the pooled leaves of five trees sampled in the spring, summer, and autumn, and winter of 2014, respectively. Intervals over the bars represent the standard error of the means, whereas different letters over the bars represent statistically different values at *p* ≤ 0.05 (Tukey–Kramer test). DW = dry weight.

**Figure 6:**
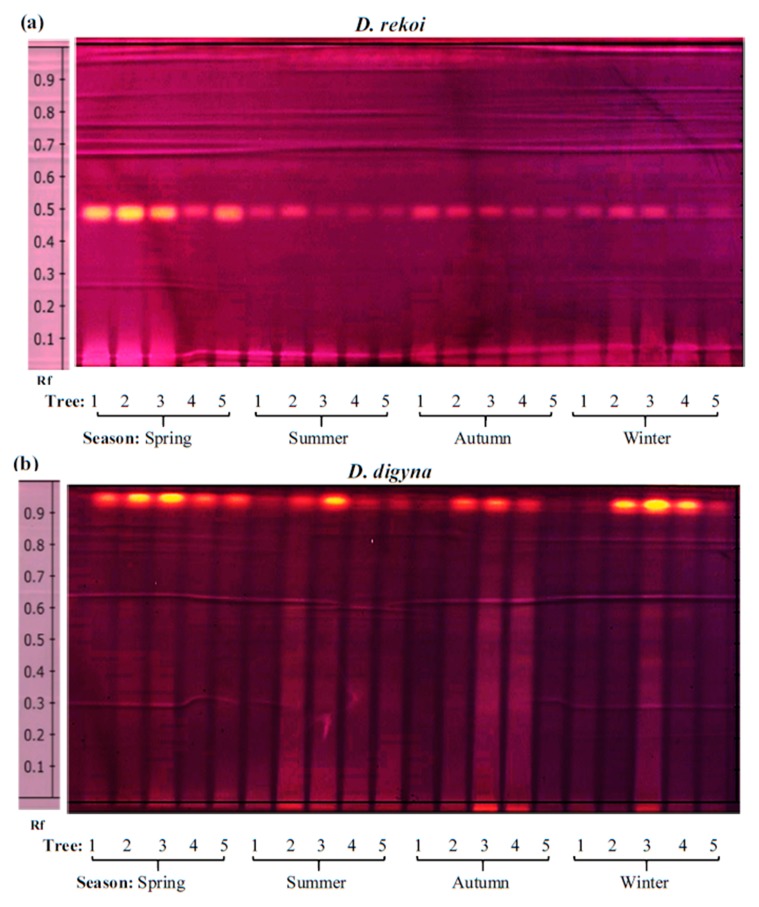
Antioxidant activity in *D. rekoi* and *D. digyna* leaf extracts. Bands showing antioxidant activity using the DPPH reagent were detected in polar leaf extracts previously separated on HP-TLC plates. The aqueous methanol extracts were obtained from leaves of five (**a**) *D. rekoi* and (**b**) *D. digyna* trees (Trees: 1–5) that were systematically sampled in the spring, summer, autumn, and winter of 2014, respectively. Band Rf values are represented on the left-side end of the figures.

The results shown in Figure 5 showed that, compared to *D. rekoi* LEs, antioxidant capacity (AOC) was significantly higher in *D. digyna* LEs, except in the spring. The significantly higher AOC detected in spring *D. rekoi* LEs, in contrast to equivalent LEs from the other seasons, was in accordance with their significant TSP and TF accumulation (Figure 3). Likewise, the antioxidant-active bands detected in *D. rekoi* LEs by HP-TLC-DPPH (Figure 6a) were stronger in the spring and migrated in a zone characterized by the presence of yellow–orange bands, usually representative of flavonoid glycosides considered to have relevant antioxidant activity [18]. On the other hand, DPPH-HPLTC traces showed that antioxidant bands produced by *D. digyna* LEs tended to be more intense in the spring and winter seasons (Figure 6b). The intensity of antioxidant-activity positive bands was also observed to vary between LEs of trees sampled within the same season. A densitometric analysis that quantified the total band intensity present in each individual DPPH-TLC trace confirmed the tendencies described above (Table S2).

No antifungal activity against two known phytopathogenic species was detected in *Diospyros* leaf extracts (results not shown), while a moderate activity against phytopathogenic Gram-positive and Gram-negative bacteria was observed in *D. rekoi* and *D. digyna* LEs (Table 3). Growth inhibition zones did not extend beyond 20 mm and were frequently independent of the volume applied. The antibacterial effect observed against *Clavibacter michiganensis* (*Cmm*) was apparently species- and season-specific, since inhibitory activity was only observed in extracts obtained from spring and summer LEs derived from *D. rekoi* and *D. digyna* trees, respectively.

## 3. Discussion

The HP-TLC metabolic fingerprints generated by LEs of two contrasting *Diospyros* species were clearly different. While LE fingerprints of *D. rekoi* showed great band diversity and seasonal variability, those from *D. digyna* were more homogeneous and were less influenced by seasonality. Although less evident, a certain degree of leaf metabolic variability in the trees sampled was also observed. A previous study implied that domestication of *D. digyna* led to the production of larger and more symmetrical fruits, in addition to a divergence from *D. rekoi* fruits in several other physicochemical properties [16]. This concept was supported by the decreased metabolic diversity and seasonal variability in the chemistry of *D. digyna* leaves herein reported. This difference lends supports to the oft-disputed argument that domestication may lead to a loss of genetic diversity [19,20] and is in agreement with number of studies that revealed significant domestication-associated metabolomic changes in certain crop plants. Recent illustrative examples include strawberry [21], tomato [22], sweet potato [23], wheat [24], and maize [20].

Likewise, field experimental data [15] suggested that the seasonal variations in leaf secondary metabolite content observed could be explained also by different patterns of carbon mobilization, which were associated with their deciduous (i.e., *D. rekoi*) or evergreen (i.e., *D. digyna*) leaf persistence habit and/or as a response to more stressful conditions faced by *D. rekoi* trees in contrast to the more controlled conditions in which *D. digyna* trees were cultivated. Seasonal changes in temperature, photoperiod, and/or water and nutrients availability recorded in the contrasting sampling sites could have been additional factors influencing leaf metabolism in leaves of *D. digyna* and *D rekoi* (see below). In this respect, the accumulation of abundant organic acids in LEs of *D. rekoi* coincided with copious experimental evidence linking plant organic acid metabolism with tolerance to environmental stresses and other detrimental ambient factors, including soil nutrient deficiencies [25]. Other ontogenetic factors associated with the deciduous or evergreen physiology of these *Diopsyros* tree species could have been instrumental for their distinct seasonal changes in plant metabolite profiles [26]. Emblematic examples of seasonal and/or ontogenetic changes in the accumulation of phenolic compounds similar to those found in *D. rekoi* LEs have been reported before in oak [27,28] and *Geranium sylvaticum* [29].

The presence of prominent yellow–orange bands in leaf extracts of both *Diospyros* species, which were localized in the Rf ≈ 0.2–0.8 range was reminiscent of data from previous HP-TLC analysis of LEs of pharmaceutically relevant plants [30], where similar yellow spots were tentatively identified as flavonoid quercetin glycosides [31,32,33]. Such a proposal is supported by the results shown in Table 1 which indicated a differential abundance of quercetin and quercetin 3-O glucuronides in *D. rekoi* LEs. It also coincided with the chemical composition of *D. kaki* leaves, where quercetin was found to be one of the main chemical constituents [13]. A prominent blue band (Rf ≈ 0.25) was also detected in the majority of *D. rekoi* leaf extracts, comparable to an unidentified and intense band (Rf ≈ 0.22) formerly reported in cultivated and wild garlic leaf extracts [30]. Its position in the HP-TLC plate, together with its characteristic blue coloration, suggest that it could correspond either to a phenolic acid or a coumarin, similarly to the blue–green bands observed to migrate from an Rf ≈ 0.2 upwards in *D. rekoi* and, to a lesser degree, in *D. digyna* LEs [31,33]. This possibility is sustained by the exclusive detection by GC-MS (Table 2) of p-coumaric and ferulic acids, in addition to a higher accumulation of p-hydroxybenzoic acid, in *D. rekoi* LEs which was in correspondence with the high proportion of p-coumaric and ferulic acids in the phenolic composition of *D. kaki* leaves [34]. These two phenolic compounds could have also contributed to the AOC detected in their respective LEs, as reported previously in fruits of six *D. kaki* phenotypes, whose AOC was similarly analyzed using the FRAP and DPPH methods [35]. Curiously, these three compounds were also reported as contributors of AOC in *D. digyna* fruits [6], although p-hydroxybenzoic acid was shown to have a negative correlation with AOC in *D. kaki* fruits [35]. However, the detection of relatively high levels of 6, 7-dihydroxycoumarin (or esculetin) in *D. digyna* LEs (Table 2) strongly suggested that it was a main contributor to their AOC. This proposal is sustained by data showing that this phenolic compound was among the coumarins having the highest AOC, a property mostly associated with its ability to deplete DPPH, peroxy, and superoxide ion radicals [36,37,38].

The detection of AOC in *D. rekoi* and *D. digyna* LEs conformed to reports describing AOC in proanthocyanidin-rich *D. kaki* leaf extracts [13] and in gallic acid- and myricetin-abundant extracts of *D. lotus* fruits [39]. They also agreed with free radical scavenging effects observed in extracts of *D. malabarica* [9,13] and with reduced lipid peroxidation via the inhibition of soybean lipoxygenase 15 in *D. abyssinica*, [40]. Likewise, this study indicated that the AOC detected in the *Diospyros* LEs analyzed, not only varied among species but also among individual trees within a single season. A possible explanation for this behavior could be related to previous findings reporting that phenolic-like AOC in plants can be affected by several factors aside from domestication and genotype, such as growth stage, leaf position, and time of harvesting [41,42,43]. Nevertheless, the results derived from this study suggest that along with *D. kaki* leaf infusions, leaves of Mesoamerican *Diospyros* species can be considered as additional sources of antioxidant-rich properties.

Similar to what was observed in *D. rekoi* and *D. digyna*, p-coumaric and p-hydroxybenzoic acid were also found to vary seasonally in fruits of *D. lotus* [44]. Moreover, p-hydroxybenzoic acid, also present in several other plant sources [45], could contribute to 1,4-naphthoquinone biosynthesis in these two *Diospyros* trees, similarly to what was previously reported in certain plants of the Boraginaceae family [46]. A role in defense is also suggested, based on the observed accumulation of p-hydroxybenzoic acid and salicylic acid in the phloem of cucumber plants undergoing systemic acquired resistance [47]. Apropos of naphthoquinones, they are important secondary metabolites having therapeutic value that are commonly found in various *Diospyros* species [48]. In this context, the detection of the trigonelline–nicotinic acid combination, only detected in *D. rekoi* LEs was also interesting, considering that trigonelline, a pyridine alkaloid derived from nicotinic acid [49], has been reported to have a potent anti-diabetic effect, similar to naphthoquinones [50,51]. Approximately 100 trigonelline-forming plant species have been identified, including *D. mollis* [49,52].

The defined season- and species-specific anti-*Cmm* activity pattern observed (Table 3) suggests either the presence of species-specific active compound(s) or shared active compound(s) whose abundance in leaves of each *Diospyros* species could have been differentially influenced by season. In contrast, the effect again *Pst* remained largely unaffected by concentration, season, and species. The effect that seasonality might have in the antimicrobial potential of polar and/or non-polar extracts obtained from different organs and/or tissues of several other *Diospyros* species has not been documented. However, the effect observed could be partly explained by the demonstrated influence that (a)biotic factors, such as growing conditions, temperature, light, nutrients, water, etc., in addition to differential responses to these factors observed among species or cultivars, have on the chemical composition of plants. Such changes directly impact the abundance of secondary metabolites known to have protective and/or beneficial uses for humans [53]. An illustrative example, among many, is the geographical and seasonal variation reported in the antimicrobial activity of various South African medicinal plant species [54]. On the other hand, the modest antibacterial effect observed was partly in agreement with several reports describing similar activity, mostly against food-spoilage microorganisms and food-borne and human pathogens, in leaf extracts of various *Diopyros* species [9,13,55,56]. Triterpenoids, coumarins, and flavonoids, some of which were identified in *D. rekoi* and *D. digyna* LEs, have been reported as possible antibacterial compounds, although the weak effect observed could have been due to the absence of non-polar naphthoquinones, considered to have high antibacterial activity [13,55,57,58]. The lack of antifungal activity detected in *D. digyna* and *D. rekoi* LEs was contrary to a number of reports noting antifungal activity in *Diospyros* extracts of diverse nature and origin [9,59,60]. Again, this absence could be partially explained by the use of polar solvents unable to extract the highly biocidal but non-polar naphthoquinones. Nevertheless, the lack of antifungal activity was contrary to the detection of phenolic compounds in the LEs of these two *Diospyros* species, considering that several phenolic compounds have been found to be effective against fungi and yeast, presumably by disrupting mitochondrial oxidative phosphorylation [58,61]. This apparent contradiction will require further experimentation to be resolved.

## 4. Materials and methods

### 4.1. Biological Material

Leaf samples from five individual *D. digyna* and *D. rekoi* trees per sampling site, respectively, were obtained in two different locations in western Mexico as previously described [15]. Sampling was done within a 16-month span that started in November of 2013 and concluded in February of 2015. Two bacterial plant pathogens were employed in this study. The Gram-positive *Clavibacter michiganensis* subsp. *michiganensis* (*Cmm*), Arista strain, was generously provided by A. Alpuche (IPCyT, San Luis Potosí, México). It was grown in yeast–dextrose–agar (yeast nutrient broth, 0.8%; yeast extract, 0.2%; K_2_HPO_4_, 0.2%; KH_2_PO_4_, 0.025%; agar, 1.5%). The Petri dishes were incubated at 28 °C until the surface was completely covered by a continuous bacterial film. On the other hand, the Gram-negative *Pseudomonas syringae* pv. *tomato* strain DC3000 (*Pst*) was kindly provided by Alejandro Peñaloza (Department of Entomology and Plant Pathology, Oklahoma State University). *Pseudomonas syringae* pv. *tomato* was maintained on King’s broth media [62] supplemented with nalidixic acid as described previously [63]. Aliquots (300 µl) were taken from the tubes and plated on mannitol-glutamate media, pH 7.0 [64]. Petri dishes were also incubated at 28 °C until the surface was completely covered by a continuous bacterial film. The plant fungal pathogens employed were *Fusarium oxysporum* f. sp. *ciceris*, generously donated by Ernestina Valadez-Moctezuma, (Universidad Autónoma Chapingo, Mexico) and *Colletotrichum gloeosporiodes* (ATCC MYA 456), donated by Juan José Peña Cabriales (Cinvestav, Irapuato). Both isolates were cultivated on potato dextrose agar for 96 h at 25 °C in the dark. When required, spore suspensions of both phytopathogenic fungi were prepared, in 0.01% Triton X-100, from sporangia harvested from 10 day cultures maintained at 28 °C.

### 4.2. Preparation of Leaf Extracts

*Diospyros* leaves were flash frozen in liquid nitrogen as described in Reference [15]. Foliar material from each individual tree (i.e., 8 leaves per tree, sampled from the 4 cardinal points) was pooled, lyophilized, and finely ground in a Retsch Mixer Mill MM 400 (Verder Scientific GmbH & Co. KG; Haan, Germany) for 12 s at 30 Hz. Portions (25 mg) of plant powder were extracted with 1 mL of 60% aqueous (aq.) MeOH or EtOH with constant stirring at 200 rpm for 24 h at room temperature. The leaf extracts (LEs) were filtered through Whatman #41 filter paper directly into amber glass vials and then sealed with plastic paraffin film (Parafilm; Bemis North America, Neenah, WI, USA) and stored at 4°C until analysis. Lyophilized plant tissue was maintained at room temperature in a desiccator with lid, from which it was periodically taken, as needed, to prepare LEs that were used for the following assays: HP-TLC fingerprinting, in vitro total soluble phenols (TSP), and total flavonoids (TFs) determinations, and on-plate antioxidant and antimicrobial activity. For the UPLC-MS and GC-MS analysis, equivalent amounts of lyophilized leaf tissue of the five *D. digyna* and *D. rekoi* trees sampled in each season of 2014, were pooled together into a single sample and analyzed as described below.

### 4.3. Metabolic Fingerprinting and Antioxidant Activity by HP-TLC

Aqueous MeOH leaf extracts separated by HP-TLC were utilized to obtain metabolic fingerprints and to identify the antioxidant-active bands present in the leaf extracts of the two *Diospyros* species analyzed. Both assays were performed following a modified procedure [15] of the original methods reported by Hosu et al. [65]. The metabolic fingerprints were generated by placing the HP-TLC plates under UV light (254 and 366 nm) and under visible light for 15, 30, and 60 min after dipping the plates in “natural products (NPs)”/polyethylene-glycol solutions. The antioxidant activity evaluation was performed in plates that were immersed, after separation, in a 0.2% MeOH 1, 1-diphenyl-2-picrylhydrazyl (DPPH) solution. Antioxidant activity was revealed by pale yellow bands on a purple background. All plates were documented using a TLC visualizer device, as described before [15]. Three TLC plates per assay were employed to confirm data reproducibility. All chemicals were acquired from Sigma–Aldrich Chemicals (St. Louis, MO, USA).

### 4.4. Total Soluble Phenol (TSP) and Flavonoid (TF) Content In Vitro

The TSP and TF contents in leaves were determined as described previously by Maranz et al. [66] and Sakanaka et al. [67], respectively.

### 4.5. In Vitro Antioxidant Activity

In vitro antioxidant activity in MeOH leaf extracts was measured by means of the 2, 2-diphenyl-1-picrylhydrazyl (DPPH) and ferric ion-reducing antioxidant power (FRAP) assays, respectively, according to the methodologies described by Yahia et al. [6]. Semi-quantitative evaluation of the antioxidant activity evaluation was determined 30 min after DPPH treatment, by adding the total areas of the reactive bands which were normalized to correct for the background noise produced by variables such as layer quality and non-uniformity of the redox reaction. All plates were documented using a TLC visualizer device, as described previously [15].

### 4.6. Metabolomic Analysis of Diospyros Leaves

An untargeted metabolomic analysis of more than 900 different ionizable molecules obtained from *D. digyna* and *D. rekoi* leaf extracts was obtained by direct-injection electrospray ionization mass spectrometry (DIESI-MS) of aq. MeOH extracts as described previously [68]. Additionally, a targeted metabolomic analyses of pooled *D. digyna* and *D. rekoi* aq. EtOH leaf extracts collected in each season of 2014 (GC-MS) and aq. MeOH extracts obtained from leaves sampled in the spring of 2014 (UPLC-MS) were performed. The GC-MS analysis was performed using a DB1MS UI capillary column (60 m × 250 µm × 0.25 µm; Agilent Technologies, Santa Clara; CA, USA) fitted to a 7890A gas chromatograph coupled to a selective mass detector (Agilent Technologies). The separation conditions were the following: initial oven temperature of 70 °C, kept for 5 min and then increased to 280 °C at a rate of 5 °C/min. The injector temperature was 230 °C and the mobile phase used was helium gas flowing at a rate of 1 mL/min. Compound metabolite identification was performed using the deconvolution AMDIS program (National Institute of Standards and Technology, NIST; Gaithersburg, MD, USA) and the NIST MS Database, search version 2.0. Three technical replicates per leaf extract were included in the analysis.

For UPLC-MS analysis, aq. MeOH extracts were dried under vacuum and reconstituted in a mixture of acetonitrile/de-ionized water (20: 80, v/v) and filtered through a 0.2 μm filter. Compounds were separated on an Acquity Class I, UPLC System (Waters, Milford, MA, USA) using a CSH C_18_ 2.1 m × 150 mm, 1.7 µm; column thermo-stated at 40 °C. Sample injection volumes were 10 μL. Elution was performed at a flow rate of 0.3 mL/min with gradient separation progressing as follows: mobile phase A: de-ionized water containing 0.1% formic acid; mobile phase B: acetonitrile, containing 0.1% formic acid. The elution was isocratic for the first minute at 99% A: 1% B. Then, a 35 min linear gradient increase to 100% B was executed, followed by a 5 min hold at this concentration and a return to the initial 99% A: 1% B conditions in 5 min, for column re-equilibration. The mass spectrometer comprised an orthogonal QTOF Synapt G1 (Waters) operated under the following conditions: electrospray ionization in positive mode, capillary voltage at 3.0 kV, cone voltage 46 V, extractor voltage 4.0 V, with source and desolvation temperatures of 120 °C and 300 °C, respectively. Cone and desolvation gas flow were nitrogen at a flow rate of 20 L/h and 800 L/min, respectively. Leucine-enkephalin (M + H)^+^ = 556.2771 was infused at a flow rate of 5 μL/min and at a concentration of 2 ng/mL during data acquisition as an internal mass standard to correct for mass drift.

### 4.7. Antimicrobial Activity of Leaf Extracts

An agar disk-diffusion method [69] was employed to test for antimicrobial activity in the *Diospyros* leaf extracts. Briefly, Petri dishes prepared with the appropriate media were uniformly inoculated with a standardized concentration of the test microorganisms (i.e., 150 µL of a suspension of bacterial cells or fungal spores adjusted to a titer of 10^6^ cfu/mL). Three sterile Whatman #1 filter paper discs, 1 cm in diameter, and impregnated with 50, 100, and 200 µL of the respective leaf extracts were placed symmetrically on the agar surface. A fourth control disc contained 60% aq. MeOH only. Then, the Petri dishes were incubated under suitable conditions (i.e., 37 °C for 24 h, for bacteria, or 25 °C for 72 h, for fungal spores). Biological activity was determined by measuring the capacity of the diffused solutions to inhibit growth of the test microorganism. The extent of the antimicrobial activity was equivalent to the diameter of the inhibition haloes.

### 4.8. Data Analysis

The antioxidant and biological activity data are represented as the mean ± SD of three independent experiments (*n* = 3). The antioxidant activity was subjected to an a posteriori Tukey test to determine the statistical significance of the data at a *p* = 0.05 level. Three technical replicates of pooled *D. digyna* and *D. rekoi* spring leaf extracts were analyzed by UPLC-MS as described above. Data were first imported into Progenesis QI (small molecules software, Non-Linear Dynamics, Waters, UK), for automatic alignment, normalization, deconvolution, and compound pre-identification over all sample runs. Compounds were putatively annotated by using Chemspider Databases that comprised an online search using PlantCyc, Plant Metabolic Network, KEGG, and ChEBI. Additional data were produced by targeted MS/MS of several features found relevant by statistical analysis (see Supplemental Materials). Statistics and graphics were performed using EZinfo 3.0 and R (3.3.3v) software. The resulting data was submitted for principal component analysis (PCA) and orthogonal projections to latent structures discriminant analysis (OPLS-DA). The GC-MS-generated data were analyzed as described above.

## 5. Conclusions

This study indicated that, compared to *D. digyna*, the leaf chemistry of *D. rekoi* trees was richer, more diverse, and influenced by seasonality. The differences observed can be attributed, in part, to the domestication syndrome associated with reduced natural variation, as evidenced in several other crops. Active compound(s) responsible for leaf AOC, appeared to be species-specific. The higher AOC detected in *D. digyna* LEs may have been due to the accumulation of esculetin, known to be a very efficient scavenger of free radicals. The AOC was dependent on the season, particularly in *D. rekoi*, and varied among individual trees. Contrary to other *Diospyros* species, LEs of these Mesoamerican species had limited to nil antimicrobial activity.

The information herein reported should stimulate a more profound analysis of these two highly contrasting species, together with other poorly known Mesoamerican *Diospyros* species. Increased knowledge about these marginal species could reveal further metabolic traits that could be utilized for the improvement of commercial *Diospyros* species, such as *D. kaki.* In addition, it might promote a more organized and rational exploitation/conservation of wild and semi-domesticated Mesoamerican *Diospyros* species.

## Figures and Tables

**Table 1:** Exact mass and most important discriminant metabolites between *D. digyna* and *D. rekoi* leaves after OPLS-DA. The intensities and factor of change are based on the average of the measured values for each exact mass in the group.

m/z (M + H)^+^	Retention Time (min)	Pre-Identification	*P*(1) ^1^	*p* (corr)(1) ^1^	*p* (*D. digyna*/*D. rekoi*) ^1^	Fold-Change Factor	Average (*D. digyna*)	Average (*D. rekoi*)	SD (*D. digyna*)	SD (*D. rekoi*)
179.034	12.14	4, 7-Dihydroxy coumarin	−0.155	−1.000	1.10 × 10^−6^	239.3	56.408	0.236	2.015	0.048
303.05	14.76	Quercetin	0.158	0.999	3.0 × 10^−7^	17.8	3.471	61.922	0.219	1.504
479.082	14.76	Quercetin 3-O-glucuronide	0.162	0.999	1.34 × 10^−6^	20.2	3.206	64.837	0.348	2.298
287.055	15.35	3,4,5,7,3′,4′,5′-Heptahydroxyflavan	0.156	1.000	5.17 × 10^−8^	17.3	3.512	60.795	0.149	0.945
463.088	15.35	Quercetin-3′-glucuronide	0.152	1.000	4.58 × 10^−8^	20.9	2.734	57.056	0.038	0.879
485.33	19.17	Triterpene 1	−0.135	−0.999	2.43 × 10^−6^		42.739	0.000	1.870	0.000
469.33	21.91	Triterpene 2	−0.142	−1.000	9.97 × 10^−7^		47.091	0.000	1.648	0.000
593.28	33.19	Unidentified	0.215	1.000	4.30 × 10^−8^	10.0	12.123	121.126	0.529	1.655
621.31	35.29	Unidentified	0.197	0.995	3.38 × 10^−5^	6.8	15.792	107.537	0.885	7.722
736.54	36.85	Unidentified	0.164	0.998	2.98 × 10^−6^	121.2	0.525	53.596	0.269	2.892

^1^ Represent the covariance *p*(1) and correlation *p* (corr)(1) loadings for a two class OPLS-DA model (i e., *D. rekoi* versus *D. digyna*). *p* are pairwise *p*-values from ANOVA.

**Table 2:** Metabolites identified by a targeted metabolomic GC-MS analysis of *Diospyros* leaf extracts. Metabolites detected in leaf extracts of *D. rekoi* and *D. digyna* trees sampled in each season of 2014. Each extract was prepared with leaves of 5 individual trees per sampling site per season.

**(a) *Diospyros rekoi***							
**SPRING ^1^**		SUMMER		AUTUMN		WINTER	
Compound	% Area ^2^	Compound	% Area	Compound	% Area	Compound	% Area
Glycerol	3.7	Glycerol	1.9	Glycerol	4.02	Glycerol	2.99
dl-malic acid	3.46	Palmitic acid	0.69	dl-Malic acid	2.91	dl-Malic acid	2.94
Trigonelline	2.17	R-3-Hydroxy-butyric acid	0.560	Glyceric acid	1.51	Palmitic acid	1.45
Palmitic acid	1.65	2-Hydrxy-3-methyl-butyric acid	0.417	l-Threonic acid	1.43	Glyceric acid	1.03
l-Threonic acid	1.587	Malonic acid	0.360	Trigonelline	1.03	l-Threonic acid	0.87
Glyceric acid	1.383	Linoleic acid	0.327	Nicotinic acid	0.83	Trigonelline	0.62
Nicotinic acid	0.834	Linolenic acid	0.24	Palmitic acid	0.604	Phytol	0.39
Erythritol	0.722	Oleic acid	0.178	Succinic acid	0.48	Linolenic acid	0.33
Phytol	0.574	β-Lactic acid	0.164	Phytol	0.47	Succinic acid	0.28
Succinic acid	0.565	Phytol	0.151	Erythritol	0.29	Oleic acid	0.27
Oleic acid	0.47	Pentanoic acid	0.067	2-Hydroxy-3-methyl-butyric acid	0.28	2-Hydroxy-3-methylvaleric acid	0.198
Linolenic acid	0.389	2-Pentenoic acid	0.06	p-Coumaric acid	0.24	Erythritol	0.191
p-Coumaric acid	0.352	R-3-Hydroxy-butyric acid	0.056	2-Hydroxy-3-methylvaleric acid	0.21	Linoleic acid	0.17
2-Hydroxy 3-methyl-butyric acid	0.328	Enol pyruvate	0.05	Oleic acid	0.2	p-Coumaric acid	0.16
2-Hydroxy-3-methyl-valeric acid	0.283			Linolenic acid	0.18	2-hydroxy-3-methylbutanoic acid	0.16
Linoleic acid	0.226			Linoleic acid	0.14	Nicotinic acid	0.11
Malonic acid	0.221			Enol pyruvate	0.14	3-Hydroxy-caproic acid	0.098
β-Lactic acid	0.143			p-Hydroxybenzoic acid	0.11	2-Hydroxy-isocaproic acid	0.092
Enol pyruvate	0.14			Ethylene glicol	0.082	p-Hydroxybenzoic acid	0.093
Ferulic acid	0.123			2-OH-Isocaproic acid	0.079	Enol pyruvate	0.092
2-Hexenoic acid	0.114			Malonic acid	0.069	Malonic acid	0.091
2-Hydroxy-isocaproic acid	0.109			4-Hydroxy-butanoic acid	0.065	β-lactic acid	0.08
p-Hydroxybenzoic acid	0.090			β-Lactic acid	0.049	2-Butenedioic acid	0.058
2-Butenedioic acid	0.071			Ferulic acid	0.038	Ethylene glicol	0.051
4-Hydroxy-butanoic acid	0.069			Propylene glicol	0.036	4-Hydroxy-butanoic acid	0.049
Hexanoic acid	0.047			R-3-Hydroxy-butyric acid	0.036	R-3-hydroxy-butanoic acid	0.038
Ethylene glicol	0.03			Hexanoic acid	0.035	Ferulic acid	0.036
R-3-Hydroxy-butyric acid	0.026			2-Hydroxy-2-methyl-butyric acid	0.033	Propylene glicol	0.026
Propylene glicol	0.023			2-Hexenoic acid	0.026	2-Hexenoic acid	0.022
Pentanoic acid	TR ^3^			Pentanoic acid	TR	Malonic acid	0.091
2-Pentenoic acid	TR					Pentanoic acid	TR
2-Hydroxy 2-methyl-Butyric acid	TR					2-Pentenoic acid	TR
						2-Hydroxy-2-methylbutanoic acid	TR
**(b) *Diospyros digyna***							
**SPRING ^1^**		SUMMER		AUTUMN		WINTER	
**Compound**	% Area ^2^	Compound	% Area	Compound	% Area	Compound	% Area
Glicerol	2.19	Glicerol	1.77	Glicerol	2.58	6,7-Dihydroxycoumarin	3.06
6,7-Dihydroxycoumarin	2.02	6,7-Dihydroxycoumarin	1.31	6,7-Dihydroxycoumarin	2.14	Glicerol	2.98
dl-Malic acid	1.84	dl-Malic acid	1.06	dl-Malic acid	2.16	Linolenic acid	1.94
Linolenic acid	1.52	Linolenic acid	1.1	Linolenic acid	1.73	Palmitic acid	1.29
l-Threonic acid	1.16	l-Threonic acid	0.55	Palmitic acid	1.31	dl-Malic acid	0.91
Palmitic acid	1.03	Palmitic acid	0.54	l-Threonic acid	0.98	l-Threonic acid	0.85
Glyceric acid	0.45	Glyceric acid	0.37	Glyceric acid	0.50	Glyceric acid	0.60
Oleic acid	0.27	Oleic acid	0.19	Oleic acid	0.40	Oleic acid	0.42
Linoleic acid	0.17	Linoleic acid	0.16	Linoleic acid	0.26	Linoleic acid	0.26
Nicotinic acid	0.16	Erythritol	0.13	Erythritol	0.24	Erythritol	0.16
Erythritol	0.13	Enol pyruvate	0.057	Enol pyruvate	0.08	β-Lactic acid	0.095
β-Lactic acid	0.101	β-Lactic acid	0.056	Phytol	0.072	Enol pyruvate	0.073
p-Hydroxybenzoic acid	0.06	p-Hydroxybenzoic acid	0.05	β-Lactic acid	0.07	Succinic acid	0.053
Enol pyruvate	0.049	Succinic acid	0.038	Propylene glicol	0.061	Ethylene glicol	0.046
Phytol	0.049	Phytol	0.034	Ethylene glycol	0.055	Propylene glycol	0.038
Succinic acid	0.04	Propylene glicol	0.024	Succinic acid	0.05	Propylene glicol	0.038
Ethylene glicol	0.029	Ethylene glicol	0.023	2-Butenedioic acid	0.018	Malonic acid	0.026
Propylene glicol	0.027	Nicotinic acid	0.015	Pyruvic acid	0.004	Pyruvic acid	0.015
Malonic acid	0.021	2-Butenedioic acid	0.015	2-Hydroxy-3-methylvaleric acid	TR	Nicotinic acid	0.014
Pyruvic acid	0.011	Malonic acid	0.013	2-Hydroxy-isocaproic acid	TR	2-Butenedioic acid	0.013
2-Hydroxy-3-methylvaleric acid	TR ^3^	Pyruvic acid	0.003	4-Hydroxy-butanoic acid	TR	2-Hydroxy-3-methylvaleric acid	TR
2-Hydroxy-isocaproic acid	TR	2-Hydroxy-3-methylvaleric acid	TR	2-Hydroxy-3-methylbutanoic acid	TR	2-Hydroxy-isocaproic acid	TR
4-Hydroxy-butanoic acid	TR	2-Hydroxy-isocaproic acid	TR	R-3-Hydroxy-butanoic acid	TR	4-Hydroxy-butanoic acid	TR
2-Hydroxy-3-methylbutanoic acid	TR	4-Hydroxy-butanoic acid	TR	2-Hydroxy-2-methylbutanoic acid	TR	2-Hydroxy-3-methylbutanoic acid	TR
R-3-Hydroxy-butanoic acid	TR	2-Hydroxy-3-methylbutanoic acid	TR			R-3-Hydroxy-butanoic acid	TR
2-Hydroxy-2-methylbutanoic acid	TR	R-3-Hydroxy-butanoic acid	TR			2-Hydroxy-2-methylbutanoic acid	TR
		2-Hydroxy-2-methylbutanoic acid	TR				

^1^ Leaves from the five trees sampled every season were pooled together into a single composite sample. ^2^ The “% Area” represents the mean value of three technical replicates. ^3^ TR = Traces.

**Table 3:** Antibacterial activity in leaves of two contrasting *Diospyros* tree species. Methanolic extracts were obtained from the combined leaves (*n* = 8) sampled from the canopy of five *D. digyna* and *D. rekoi* trees at different locations and seasons for the duration of 2014.

**^1^ Growth inhibition (mm)**												
*D. rekoi*												
**Bacterial pathogen**	Spring			Summer			Autumn			Winter		
	50 ^2^	100	200	50	100	200	50	100	200	50	100	200 μL
**^3^*Cmm***	0	0	0	0	13.2 ± 2.3	15.8 ± 1.5	0	0	0	0	0	0
**^4^*Pst***	14 ± 2.3 ^1^	15.8 ± 3.1	18.2 ± 2.7	14 ± 2.1	14.4 ± 1.6	15.4 ± 2.3	17.6 ± 3	17.6 ± 3	18.2 ± 3	13.6 ± 2	15 ± 2	15.4 ± 1.5
*D. digyna*												
**Bacterial pathogen**	Spring			Summer				Autumn		Winter		
	50	100	200	50	100	200	50	100	200	50	100	200 μL
***Cmm***	14.6 ± 3.4	16.1 ± 1.9	18.4 ± 3.7	0	0	0	0	0	0	0	0	0
***Pst***	14.2 ± 1.4	15.2 ± 2.6	16.4 ± 2.9	0	15 ± 1.7	15.6 ± 3.8	15.2 ± 1.2	16 ± 2.4	18.2 ± 1	0	0	0

^1^ Diameter of growth inhibition zones around the *Diospyros* leaf extracts (LEs). ^2^ Volume of LE applied. ^3^ *Cmm*: *Clavibacter michiganensis* ssp. *michiganensis*. ^4^ *Pst*: *Pseudomonas syringae* pv. *tabaci*.

## Supplementary Materials

The following are available online at https://www.mdpi.com/2223-7747/8/11/449/s1, Figure S1: PCA score plot of *D. digyna* and *D. rekoi* leaf extracts sampled in the spring of 2014, Figure S2. S-plot between *D. digyna* (=−1) and *D. rekoi* (=+1) generated with a 6 observation dataset (3 *D. digyna* and 3 *D. rekoi*), Table S1: Total number of metabolites detected by different analytical methods in leaf extracts of *Diospyros digyna* and *D. rekoi* trees sampled in different seasons of the year 2014, Table S2: In vitro antioxidant capacity in leaves of two contrasting *Diospyros* tree species.

## References

[1] Leonti M., Cabras S., Castellanos M.A., Challenger A., Gertsc J., Casu L. (2013). Bioprospecting: Evolutionary implications from a post-olmec pharmacopoeia and the relevance of widespread taxa. J. Ethnopharmacol..

[2] Provance M.C., García-Ruiz I., Thommes C., Ross-Ibarra J. (2013). Population genetics and ethnobotany of cultivated *Diospyros riojae* Gómez Pompa (Ebenaceae), an endangered fruit crop from México. Genet. Resour. Crop Evol..

[3] Pennington T., Sarukhán J. (2005). Árboles Tropicales de México. Manual Para la Identificación de las Principales Especies.

[4] Wallnöfer B. (2007). A revisión of neotropical *Diospyros* (Ebenaceae) part 1. Ann. Nat. Mus. Wien.

[5] García-Díaz R., Cuevas J.A., Segura S., Basurto F. (2015). Panbiogoegraphic analysis of *Diospyros* spp. (Ebenaceae) in Mexico. Rev. Mex. Cienc. Agric..

[6] Yahia E.M., Gutierrez-Orozco F., Arvizu-de León C. (2011). Phytochemical and antioxidant characterization of the fruit of black sapote (*Diospyros digyna* Jacq.). Food Res. Int..

[7] Peyrat L., Eparvier V., Eydoux C., Guillemot J.C., Stien D., Litaudon M. (2016). Chemical diversity and antiviral potential in pan tropical *Diospyros* genus. Fitoterapia.

[8] Peyrat L.A., Eparvier V., Eydoux C., Guillemot J.C., Litaudon M., Stien D. (2017). Betulinic acid, the first lupane-type triterpenoid isolated from both a *Phomopsis* sp. and its host plant *Diospyros carbonaria* Benoist. Chem. Biodivers..

[9] Rauf A., Uddin G., Patel S., Khan A., Halim S.A., Bawazeer S., Ahmad K., Muhammad N., Mubarak M.S. (2017). *Diospyros*, an under-utilized, multi-purpose plant genus: A review. Biomed. Pharmacother..

[10] Maroyi A. (2018). *Diospyros lycioides* Desf.: Review of its botany, medicinal uses, pharmacological activities and phytochemistry. Asian Pac. J. Trop. Biomed..

[11] Pérez-Burillo S., Oliveras M.J., Quesada J., Rufián-Henares J.A., Pastoriza S. (2018). Relationship between composition and bioactivity of persimmon and kiwifruit. Food Res. Int..

[12] Mallavadhani U., Panda A., Rao Y. (1998). Pharmacology and chemotaxonomy of *Diospyros*. Phytochemistry.

[13] Xie C., Xie Z., Xu X., Yang D. (2015). Persimmon (*Diospyros kaki*) leaves: A review on traditional uses, phytochemistry and pharmacological properties. J. Ethnopharmacol..

[14] Moo-Huchin V.M., Estrada-Mota I., Estrada-León R., Cuevas-Glory L., Ortiz-Vázquez E., Vargas y Vargas M.L., Betancur-Ancona D., Sauri-Duch E. (2014). Determinations of some physicochemical characteristics, bioactive compounds and antioxidant activity of tropical fruits from Yucatán, México. Food Chem..

[15] Ramírez-Briones E., Rodríguez-Macías R., Salcedo-Pérez E., Martínez-Gallardo N., Tiessen A., Molina-Torres J., Délano-Frier J., Zañudo-Hernández J. (2017). Seasonal variation in non-structural carbohydrates, sucrolytic activity and secondary metabolites in deciduous and perennial *Diospyros* species sampled in Western Mexico. PLoS ONE.

[16] Ramírez-Briones E., Rodríguez Macías R., Casarrubias-Castillo K., del Río R.E., Martínez-Gallardo N., Tiessen A., Ordaz-Ortiz J., Cervantes-Hernández F., Délano-Frier J.P., Zañudo-Hernández J. (2019). Fruits of wild and semi-domesticated *Diospyros* tree species have contrasting phenological, metabolic, and antioxidant activity profiles. J. Sci. Food Agric..

[17] Provance M.C., García Ruiz I., Sanders A.C. (2008). The *Diospyros salicifolia* complex (Ebenaceae) in Mesoamerica. J. Bot. Res. Inst..

[18] Falcone Ferreyra M.L., Rius S.P., Casati P. (2012). Flavonoids: Biosynthesis, biological functions, and biotechnological applications. Front. Plant. Sci..

[19] Harrigan G.G., Martino-Catt S., Glenn K.C. (2007). Metabolomics, metabolic diversity and genetic variation in crops. Metabolomics.

[20] Flint-Garcia S.A. (2013). Genetics and consequences of crop domestication. J. Agric. Food Chem..

[21] Vallarino J.G., de Abreu E., Lima F., Soria C., Tong H., Pott D.M., Willmitzer L., Fernie A.R., Nikoloski Z., Osorio S. (2018). Genetic diversity of strawberry germplasm using metabolomic biomarkers. Sci. Rep..

[22] Zhu G., Wang S., Huang Z., Zhang S., Liao Q., Zhang C., Lin T., Qin M., Peng M., Yang C. (2018). Rewiring of the fruit metabolome in tomato breeding. Cell.

[23] Drapal M., Rossel G., Heider B., Fraser P.D. (2019). Metabolic diversity in sweet potato (*Ipomoea batatas*, Lam.) leaves and storage roots. Hort. Res..

[24] Chandrasekhar K., Shavit R., Distelfeld A., Christensen S.A., Tzin V. (2018). Exploring the metabolic variation between domesticated and wild tetraploid wheat genotypes in response to corn leaf aphid infestation. Plant. Signal. Behav..

[25] López-Bucio J., Nieto-Jacobo M.F., Ramírez-Rodríguez V., Herrera-Estrella L. (2000). Organic acid metabolism in plants: From adaptive physiology to transgenic varieties for cultivation in extreme soils. Plant Sci..

[26] Koricheva J., Barton K., Iason G.R., Dicke M., Hartley S. (2012). Temporal changes in plant secondary metabolite production: Patterns, causes and consequences. The Ecology of Plant Secondary Metabolites (From Genes to Global Processes).

[27] Salminen J.P., Ossipov V., Haukioja E., Pihlaja K. (2001). Seasonal variations in the content of hydrolysable tannins in leaves of *Betula Pubescens*. Phytochemistry.

[28] Salminen J.P., Roslin T., Karonen M., Sinkkonen J., Pihlaja K., Pulkkinen P. (2004). Seasonal variation in the content of hydrolyzable tannins, flavonoid glycosides and proanthocyanidins in oak leaves. J. Chem. Ecol..

[29] Tuominen A., Salminen J.P. (2017). Hydrolyzable tannins, flavonol glycosides, and phenolic acids show seasonal and ontogenic variation in *Geranium sylvaticum*. J. Agric. Food Chem..

[30] Bubueanu C., Pavaloiu R. (2016). HPTLC chromatographic polyphenolic fingerprints of plant species from Eastern Europe. Malays. J. Med. Biol. Res..

[31] Wagner H., Bladt S. (2001). Plant. Drug Analysis: A Thin Layer Chromatography Atlas.

[32] Nile S.H., Park S.W. (2014). HPTLC analysis, antioxidant and anti-gout activity of Indian plants. Iran. J. Pharm. Res..

[33] González B., Vogel H., Razmilic I., Wolfram E. (2015). Polyphenol, anthocyanin and antioxidant content in different parts of maqui fruits (*Aristotelia chilensis*) during ripening and conservation treatments after harvest. Ind. Crop. Prod..

[34] Gorinstein S., Zachwieja Z., Folta M., Barton H., Piotrowicz J., Zemser M., Weisz M., Trakhtenberg S., Màrtín-Belloso O. (2001). Comparative content of dietary fiber total phenolics and minerals in persimmon and apples. J. Agric. Food Chem..

[35] Pu F., Ren X., Zhang X. (2013). Phenolic compounds and antioxidant activity in fruits of six *Diospyros kaki* genotypes. Eur. Food Res. Technol..

[36] Kim N.M., Kim J., Chung H.Y., Choi J.S. (2000). Isolation of luteolin 7-O-rutinoside and esculetin with potential antioxidant activity from the aerial parts of *Artemisia Montana*. Arch. Pharm. Res..

[37] Vianna D.R., Bubols G., Meirelles G., Silva B.V., da Rocha A., Lanznaster M., Monserrat J.M., Garcia S.C., von Poser G., Eifler-Lima V.L. (2012). Evaluation of the antioxidant capacity of synthesized coumarins. Int. J. Mol. Sci..

[38] Šeršeň F., Lácová M. (2015). Antioxidant activity of some coumarins. Acta Fac. Pharm. Univ. Comen..

[39] Gao H., Cheng N., Zhou J., Wang B., Deng J., Cao W. (2014). Antioxidant activities and phenolic compounds of date plum persimmon (*Diospyros lotus* L.) fruits. J. Food Sci. Technol..

[40] Maiga A., Malterud K.E., Diallo D., Paulsen B.S. (2006). Antioxidant and 15-lipoxygenase inhibitory activities of the Malian medicinal plants *Diospyros abyssinica* (Hiern) F. White (Ebenaceae), *Lannea Velutina*, A. Rich (Anacardiaceae) and *Crossopteryx febrifuga* (Afzel) Benth. (Rubiaceae). J. Ethnopharmacol..

[41] Sivaci A., Duman S. (2014). Evaluation of seasonal antioxidant activity and total phenolic compounds in stems and leaves of some almond (*Prunus amygdalus* L.) varieties. Biol. Res..

[42] Vagiri M., Conner S., Stewart D., Andersson S.C., Verrall S., Johansson E., Rumpunen K. (2015). Phenolic compounds in blackcurrant (*Ribes nigrum* L.) leaves relative to leaf position and harvest date. Food Chem..

[43] Ben Ahmed Z., Yousfi M., Viaene J., Dejaegher B., Demeyer K., Mangelings D., Vander Heyden Y. (2017). Seasonal, gender and regional variations in total phenolic, flavonoid, and condensed tannins contents and in antioxidant properties from *Pistacia atlantica* ssp. leaves. Pharm. Biol..

[44] Ayaz F.A., Kadıoğlu A. (1997). Changes in phenolic acid contents of *Diospyros lotus* L. during fruit development. J. Agric. Food Chem..

[45] Khadem S., Marles R.J. (2010). Monocyclic phenolic acids; hydroxy- and polyhydroxybenzoic acids: Occurrence and recent bioactivity studies. Molecules.

[46] Widhalm J.R., Dudareva N. (2015). A familiar ring to it: Biosynthesis of plant benzoic acids. Mol. Plant..

[47] Smith-Becker J., Marois E., Huguet E.J., Midland S.L., Sims J.J., Keen N.T. (1998). Accumulation of salicylic acid and 4-hydroxybenzoic acid in phloem fluids of cucumber during systemic acquired resistance is preceded by a transient increase in phenylalanine ammonia-lyase activity in petioles and stems. Plant Physiol..

[48] Nematollahi A., Aminimoghadamfarouj N., Wiart C. (2012). Reviews on 1,4-naphthoquinones from *Diospyros* L.. J. Asian Nat. Prod. Res..

[49] Ashihara H., Yin Y., Katahira R., Watanabe S., Mimura T., Sasamoto H. (2012). Comparison of the formation of nicotinic acid conjugates in leaves of different plant species. Plant Physiol. Biochem..

[50] Rani A., Arora S., Goyal A. (2017). Antidiabetic plants in traditional medicines: A review. Int. Res. J. Pharm..

[51] Shah M.A., Keach J.E., Panichayupakaranant P. (2018). Antidiabetic naphthoquinones and their plant resources in Thailand. Chem. Pharm. Bull..

[52] Suwama T., Watanabe K., Monthakantirat O., Luecha P., Noguchi H., Watanabe K., Umehara K. (2018). Naphthalene glycosides in the Thai medicinal plant *Diospyros mollis*. J. Nat. Med..

[53] Prinsloo G., Nogemane N. (2018). The effects of season and water availability on chemical composition, secondary metabolites and biological activity in plants. Phytochem. Rev..

[54] Van Vuuren S.F. (2008). Antimicrobial activity of South African medicinal plants. J. Ethnopharmacol..

[55] Cai L., Wei G.X., Van der Bijl P., Wu C.D. (2000). Namibian chewing stick, *Diospyros lycoides*, contains antibacterial compounds against oral pathogens. J. Agric. Food Chem..

[56] Dewanjee S., Kundu M., Maiti A., Majumdar R., Majumdar A., Mandal S.C. (2007). In vitro evaluation of antimicrobial activity of crude extract from plants *Diospyros peregrine, Coccinia grandis* and *Swietenia Macrophylla*. Trop. J. Pharm. Res..

[57] Ji L.L., Zhang Q.H., Cui G.Y. (2003). Study on the antimicrobial activities of persimmon leaves against food spoilage and food-borne pathogens and related compounds. Food Sci..

[58] Rasamison V.E., Rakotondraibe H.L., Razafintsalama V., Rakotonandrasana S., Rakotondrafara A., Ratsimbason M.A., Rafidinarivo E. (2016). Chemical constituents from stems and leaves of *Diospyros gracilipes* Hiern and the antimicrobial and cytotoxic principles. J. Pharmacogn. Phytochem..

[59] Borges-Argáez R., Canche-Chay C.I., Peña-Rodríguez L.M., Said-Fernández S., Molina-Salinas G.M. (2007). Antimicrobial activity of *Diospyros anisandra*. Fitoterapia.

[60] Rashed K., Ćiríc A., Glamoclija J., Soković M. (2014). Antibacterial and antifungal activities of methanol extract and phenolic compounds from *Diospyros virginiana* L.. Ind. Crop. Prod..

[61] Morales J., Mendoza L., Cotoras M. (2017). Alteration of oxidative phosphorylation as a possible mechanism of the antifungal action of p-coumaric acid against *Botrytis cinerea*. J. Appl. Microbiol..

[62] King E.O., Ward M.K., Raney D.E. (1954). Two simple media for the demonstration of pyocyanin and fluorescin. J. Lab. Clin. Med..

[63] Valenzuela-Soto J., Maldonado-Bonilla L., Hernández-Guzmán G., Rincón-Enríquez G., Martínez-Gallardo N., Ramírez-Chávez E., Cisneros Hernández I., Hernández-Flores J.L., Délano-Frier J.P. (2015). Infection by a coronatine-producing strain of *Pectobacterium cacticidum* isolated from sunflower plants in Mexico is characterized by soft rot and chlorosis. J. Gen. Plant Pathol..

[64] Keane P.J., Kerr A., New P.B. (1970). Crown gall of stone fruit. II Identification and nomenclature of *Agrobacterium* isolates. Aust. J. Biol. Sci..

[65] Hosu A., Danciu V., Cimpoiu C. (2015). Validated HPTLC fingerprinting and antioxidant activity evaluation of twenty-seven Romanian red wines. J. Food Compos. Anal..

[66] Maranz S., Wiesman Z., Garti N. (2003). Phenolic constituents of shea (*Vitellaria paradoxa*) kernels. J. Agric. Food Chem..

[67] Sakanaka S., Tachibana Y., Okada Y. (2005). Preparation and antioxidant properties of extracts of Japanese persimmon leaf tea (kakinoha-cha). Food Chem..

[68] Palmeros-Suárez P.A., Massange-Sánchez J.A., Martínez-Gallardo N.A., Montero-Vargas J.M., Gómez-Leyva J.F., Délano-Frier J.P. (2015). The overexpression of an *Amaranthus hypochondriacus* NF-YC gene modifies growth and confers water deficit stress resistance in Arabidopsis. Plant Sci..

[69] Balouiri M., Sadiki M., Ibnsouda S.K. (2016). Methods for in vitro evaluating antimicrobial activity: A review. J. Pharm. Anal..

