# Supplementary material for: Seasonal Changes in the Metabolic Profiles and Biological Activity in Leaves of Diospyros digyna and D. rekoi “Zapote” Trees
Source: Plants (Basel). 2019 Oct 25;8(11):449. doi: 10.3390/plants8110449 (PMC6918230; doi:10.3390/plants8110449)
Supplement: Supplementary file 1 [file plants-08-00449-s001.zip › Revised Supplemental Material-01/Figure S1_R.docx]

**Figure S1.** PCA Score plot with a 6 observations (three each for *D. digyna* and *D. rekoi* leaves sampled in the spring of 2014) dataset comprised of 475 features in ESI positive ionization mode. The analysis explained 93.72% of the variance. The plot was generated after OPLS-DA with EZ information controlled by Progenesis QI 3.0.3.
