# Supplementary material for: Seasonal Changes in the Metabolic Profiles and Biological Activity in Leaves of Diospyros digyna and D. rekoi “Zapote” Trees
Source: Plants (Basel). 2019 Oct 25;8(11):449. doi: 10.3390/plants8110449 (PMC6918230; doi:10.3390/plants8110449)
Supplement: Supplementary file 1 [file plants-08-00449-s001.zip › Revised Supplemental Material-01/Figure S2_R.docx]

**Figure S2.** S-plot between *D. digyna* (= −1) and *D. rekoi* (= +1). The covariance p(1) and correlation p(corr)(1) loadings from a two class OPLS-DA model (*D. rekoi* vs. *D. digyna*) are shown here in an S-Plot format for *D. digyna*. The points are Exact Mass/ Retention Time pairs (EMRTs). The upper right quadrant of the S-plot shows those components which are elevated in *D. rekoi*, while the lower left quadrant shows EMRTs elevated in *D. digyna*. Points positioned farther along the x-axis represent a greater the contribution to the variance between the groups, while those farther up the y-axis, a higher reliability of the analytical result.
