# Supplementary material for: Seasonal Changes in the Metabolic Profiles and Biological Activity in Leaves of Diospyros digyna and D. rekoi “Zapote” Trees
Source: Plants (Basel). 2019 Oct 25;8(11):449. doi: 10.3390/plants8110449 (PMC6918230; doi:10.3390/plants8110449)
Supplement: Supplementary file 1 [file plants-08-00449-s001.zip › Revised Supplemental Material-01/Table S1_R.docx]

**Table S1. Total number of metabolites detected by different analytical methods in leaf extracts of *Diospyros digyna* and *D. rekoi* trees sampled in different seasons of the year 2014.**

| Assay/ species | SPRING | | SUMMER | | AUTUMN | | WINTER | |  | TOTAL (per year) | |
| --- | --- | --- | --- | --- | --- | --- | --- | --- | --- | --- | --- |
|  | *D. rekoi* | *D. digyna* | *D. rekoi* | *D. digyna* | *D. rekoi* | *D. digyna* | *D. rekoi* | *D. digyna* |  | *D. rekoi* | *D. digyna* |
| HP-TLC^1^ | 23 | 29 | 30 | 24 | 41 | 19 | 44 | 22 |  | 138 | 99 |
| GC-MS^2^ | 32 | 20 | 14 | 21 | 29 | 18 | 30 | 20 |  | 105 | 79 |
| U-HPLC | 8 | 10 | - | - | - | - | - | - |  | - | - |
| Total (per season) | 63 | 59 | 44 | 45 | 70 | 37 | 74 | 42 |  | | |

^1^The number represents the number of resolved bands having clearly defined Rf values.

^2^Metabolites present in trace amounts were not quantified.
