# Supplementary material for: Seasonal Changes in the Metabolic Profiles and Biological Activity in Leaves of Diospyros digyna and D. rekoi “Zapote” Trees
Source: Plants (Basel). 2019 Oct 25;8(11):449. doi: 10.3390/plants8110449 (PMC6918230; doi:10.3390/plants8110449)
Supplement: Supplementary file 1 [file plants-08-00449-s001.zip › Revised Supplemental Material-01/Table S2-R.docx]

**Table S2. *In vitro* antioxidant capacity in leaves of two contrasting *Diospyros* tree species.** Aqueous methanolic extracts were obtained from the leaf canopy (n = 8) of five *D. digyna* and *D. rekoi* trees (T1 to T5) sampled at different locations and seasons for the duration of 2015.

| TREE (T) | AREA^1^ | |
| --- | --- | --- |
|  | *D. rekoi* | *D. digyna* |
| SPRING: |  |  |
| T1 | ^a^ 13501 ± 2191^2^ | ^a^ 6190 ± 599 |
| T2 | ^a^ 10115 ± 1659 | ^a^ 6513 ± 351 |
| T3 | ^a^ 9892 ± 969 | ^a^ 6439 ± 557 |
| T4 | ^a^ 9535 ± 770 | ^a^ 6152 ± 557 |
| T5 | ^a^ 9706 ± 822 | ^a^ 6144 ± 476 |
|  |  |  |
| SUMMER: |  |  |
| T1 | ^a^ 8533 ± 975 | ^c^ 729 ± 126 |
| T2 | ^a^ 8149 ± 1150 | ^a^ 6097 ± 392 |
| T3 | ^b^ 1897 ± 332 | ^a^ 6382 ± 931 |
| T4 | ^b^ 1576 ± 567 | ^c^ 727 ± 116 |
| T5 | ^c^ 812 ± 129 | ^c^ 716 ± 48 |
|  |  |  |
| AUTUMN: |  |  |
| T1 | ^a^ 7362 ± 868 | ^c^ 677 ± 167 |
| T2 | ^b^ 3171 ± 508 | ^b^ 3473 ± 490 |
| T3 | ^b^ 3161 ± 830 | ^b^ 3056 ± 515 |
| T4 | ^b^ 3121 ± 455 | ^b^ 2636 ± 635 |
| T5 | ^b^ 2514 ± 554 | ^d^ 229 ± 54 |
|  |  |  |
| WINTER: |  |  |
| T1 | ^b^ 2487 ± 706 | ^d^ 151 ± 89 |
| T2 | ^c^ 7007 ± 868 | ^a^ 6371 ± 495 |
| T3 | ^b^ 2030 ± 442 | ^a^ 6214 ± 500 |
| T4 | ^c^ 640 ± 45 | ^a^ 6195 ± 225 |
| T5 | ^c^ 626 ± 71 | ^d^ 369 ± 89 |

^1^The area represents the mean value ± SD (n = 3) of the peak areas produced by scanning the active bands after a 30 min reaction period with DPPH.

^2^Different letters before the areas represent statistically different values at *p* ≤ 0.05.
